# HeLP: Development of occupational protection mask against surgical smoke

**DOI:** 10.1590/0034-7167-2022-0647

**Published:** 2023-11-10

**Authors:** Helenize Ferreira Lima Leachi, Renata Perfeito Ribeiro

**Affiliations:** I Universidade Estadual de Londrina. Londrina, Paraná, Brazil

**Keywords:** Smoke, Personal Protective Equipment, Air Pollutants, Occupational, Respiratory Protective Devices, Technological Development and Innovation Projects, Humo, Equipo de Protección Personal, Contaminantes Ocupacionales del Aire, Dispositivos de Protección Respiratoria, Proyectos de Desarrollo Tecnológico e Innovación, Fumaça, Equipamento de Proteção Individual, Dispositivos de Proteção Respiratória, Poluentes Ocupacionais do Ar, Projetos de Desenvolvimento Tecnológico e Inovação

## Abstract

**Objectives:**

to describe a technological innovation in the development of an individual, ergonomic, sustainable and effective occupational respiratory protection mask for workers exposed to surgical smoke.

**Methods:**

applied, exploratory, quantitative research, using design methods and tools: Sense Intent, Know Context, Know People, Frame Insights, Explore Concepts, Frame Solutions, Realize Offerings, in addition to the Product Development Process tools. It was developed from March 2019 to December 2021.

**Results:**

from the prototyping mold, it became possible to represent the abstract to the physical, where all the concepts created in the methodological steps were implemented and the necessary adjustments were made to create the model as a technological innovation, which will have the concept for product commercialization.

**Conclusions:**

a mask for protection against surgical smoke (HeLP) was developed, from the design step to the prototype development, being a technological innovation.

## RESUMO

**Objetivos:**

descrever uma inovação tecnológica do desenvolvimento de máscara de proteção ocupacional respiratória individual, ergonômica, sustentável e eficaz para trabalhadores expostos à fumaça cirúrgica.

**Métodos:**

pesquisa aplicada, exploratória com abordagem quantitativa, empregando métodos e ferramentas em *design*: *Sense Intent, Know Context, Know People, Frame Insights, Explore Concepts, Frame Solutions, Realize Offerings*, além das ferramentas do Processo de Desenvolvimento de Produtos. Foi desenvolvida no período de março de 2019 a dezembro de 2021.

**Resultados:**

a partir do molde da prototipagem, tornou-se possível a representação do abstrato para o físico, onde implementou os conceitos criados nas etapas metodológicas e realizou os ajustes necessários para a criação do modelo como uma inovação tecnológica em que terá o conceito para a comercialização do produto.

**Conclusões:**

foi desenvolvida uma máscara de proteção contra a fumaça cirúrgica (HeLP), desde a etapa de projeto até o desenvolvimento do protótipo, configurando-se uma inovação tecnológica.

## RESUMEN

**Objetivos:**

describir una innovación tecnológica en el desarrollo de una máscara de protección respiratoria ocupacional individual, ergonómica, sostenible y eficaz para trabajadores expuestos al humo quirúrgico.

**Métodos:**

investigación exploratoria aplicada con un enfoque cuantitativo, utilizando métodos y herramientas de diseño: *Sense Intent, Know Context, Know People, Frame Insights, Explore Concepts, Frame Solutions, Realize Offers*, además de las herramientas del Proceso de Desarrollo de Producto. Fue desarrollado de marzo de 2019 a diciembre de 2021.

**Resultados:**

a partir del molde de prototipado, se logró representar lo abstracto a lo físico donde implementó los conceptos creados en las etapas metodológicas y realizó los ajustes necesarios para la creación del modelo como innovación tecnológica en el cual tendrá el concepto para la comercialización del producto.

**Conclusiones:**

se desarrolló una máscara de protección contra el humo quirúrgico (HeLP), desde la etapa de diseño hasta el desarrollo del prototipo, configurando una innovación tecnológica.

## INTRODUCTION

Surgeons and operating room workers are exposed to a variety of occupational hazards, including chemical pollution in the operating room air. As an example, there are particulate matter in aerosol, present in surgical smoke, composing the list of occupational pollutants in the air^(1)^.

Surgical smoke, formed by burning tissue during electrocautery use in surgical procedures, contains chemical products in gaseous form, cell particles, bacteria and viruses^(2)^. These particles can reach the breathing zone of workers who are close to the surgical field^(3)^.

Inhalation of particulate matter by exposed professionals can cause adverse effects to the circulatory, respiratory and nervous systems, as particles of 10 µm or smaller, when inhaled, cause irritation and long-term complications^(2)^.

Each year, millions of healthcare workers, including surgeons and nurses, are exposed to surgical smoke, but there is still no regulatory consensus among international surgical and occupational health organizations to reduce the exposure of workers to particulate matter present in surgical smoke. Thus, the National Institute of Occupational Safety and Health (NIOSH), the Occupational Safety and Health Administration (OSHA), and the Association of periOperative Registered Nurses (AORN) introduced recommendations regarding Personal Protective Equipment (PPE) use, local ventilation and exhaustion as well as aspiration of surgical smoke at the place of its production^(4, 5, 6)^.

The PPE recommended for use in operating rooms during procedures that produce surgical smoke are N95 masks and goggles.

The N95 mask is tested and found to be a high filtration mask^(6)^. However, in addition to this recommendation not being a regulatory requirement and its use being limited to procedures considered infectious-contagious, there are studies that indicate that the leakage that occurs when using this mask, caused by inadequate sealing of users’ face, is the main way of penetration of surgical smoke particles. Moreover, it does not capture gases and vapors, present in surgical smoke, due to the lack of a filter for adsorption of chemical components^(7, 8)^.

However, simple surgical masks, which are not efficient, as they are not designed to filter particulate matter, are commonly used by workers in operating rooms^(3)^.

In addition to the aforementioned issues, there is low adherence to PPE use, which may be related to the inadequate mask size for each type of face, lack of resources to acquire it, discomfort during its use and mainly lack of knowledge of its preventive role^(9)^. Moreover, prolonged N95 mask use causes injuries to the skin on users’ face, due to the pressure it exerts on the skin, in addition to the fact that the material used for its production is unsuitable for skin contact^(10)^.

Considering that N95 respiratory masks present discomfort to professionals who use them and simple surgical masks as well as N95 masks provide the questioned protection against surgical smoke, it becomes important to develop an effective, ergonomic and sustainable mask for workers’ respiratory protection in operating rooms, in addition to greater accessibility for purchase.

## OBJECTIVES

To describe a technological innovation in the development of an individual, ergonomic, sustainable and effective occupational respiratory protection mask for workers exposed to surgical smoke.

## METHODS

### Ethical aspects

The present study involved the development of a product through a study of available literature. Thus, it was not necessary to be assessed by a Research Ethics Committee, since it does not involve research with human beings.

### Study design

This is applied, exploratory, quantitative research, which generates products for application in practice, aiming to solve a specific problem^(11)^, found by research carried out by the researchers’ expertise.

### Procedure

To develop a surgical smoke protection mask prototype, exploratory research was carried out using methods and tools based on design, consisting of seven steps^(12)^, which has the proposal of innovation, creating products and services, in addition to using the Product Development Process tools^(13)^. The research was carried out from March 2019 to December 2021.

The design innovation process is a process that moves from one side to the other, i.e., it is not a linear process. The lower left quadrant represents “research”, the upper left quadrant “analysis”, through which information from reality is processed in abstract terms to create the mental models that will drive innovation. The upper right quadrant is “synthesis” and the lower right quadrant is “achievement”. All these quadrants combined form a formal process model for driving innovations^(12)^.

In the description of the steps of the entire process of construction and development of a protective mask prototype, it was decided to emphasize the exploration of concepts and the structuring of solutions found at each moment of this development; therefore, the steps followed for prototype development, with the objectives and description of the activities of each step, were as follows:

**Step 1: Sense Intent** – Objective: to recognize the reality in relation to technological and societal changes, for project elaboration. This step comprises 4 substeps: Key Facts - collection of information in scientific research to justify the development of this innovation; Patent Search - patent database search to identify similar products; Trends Experts Interview - search for experts to identify trends and future product developments; Project Development – project preparation with the objectives and methodology, which will support the project.

**Step 2: Know Context** – Objective: to study the context, circumstances or events that affect the environment in which products are used in the market. This step comprises 4 substeps: Contextual Research Plain - elaboration of a detailed schedule, with the presentation of the work team, communication channels and meetings; Popular Media Research - discovering innovations made about context in the media landscape; Publications Research - conducting research of publications on the topic of interest; Innovation Evolution Map - shows the evolution of the industry over time.

**Step 3: Know People** – Objective: to understand people, end users or other stakeholders, and their interactions during daily life. This step comprises 2 substeps: Research Participant Map - helps to have an overview of all people involved in the project topic, based on their roles and activities, in order to ensure that the right people are searched; POEMS - studies the people, objects, environment, messages and services of a given context.

**Step 4: Frame Insights** – Objective: to structure what was found in the research and in the previous steps. This step comprises 3 substeps: Observations to Insights - learning from what is observed in the research, revealing meanings; Design Principles Generation - turning research insights into actionable, predictive statements to guide ideation; Analysis Workshop - conduct a working session to understand insights, find patterns and create frameworks for ideations.

**Step 5: Explore Concepts** – Objective: to identify opportunities and explore new concepts. This step comprises 5 substeps: Principles to Opportunities - transition from analysis to synthesis; Persona Definition - defines users’ personality to explore the concept around them; Behavioral Prototype - simulate user activity situations to understand behaviors and construct initial concepts; Concept Prototype - incorporate concepts into tangible forms to receive feedback from users; Concept Sketch - visualize concepts as a sketch to show how they work in abstract terms.

**Step 6: Frame Solutions** – Objective: to structure solutions. This step comprises 2 substeps: Solution Prototype - simulate experiences around proposed solutions; Synthesis Workshop -structured brainstorming method focused on generating concepts.

**Step 7: Realize Offerings** – Objective: when potential solutions are framed and prototypes tested and assessed for implementation. This step comprises 1 substep: Pilot Development and Testing - taking offers to market to learn how they work and how users experience them.

The mask prototype development steps were aligned with the Product Development Process tools^(13)^, which is the connection with the market, where the need to propose solutions is identified. Thus, the process is divided into three macro phases: pre-development, development and post-development.

The pre-development phase ensures developing the product by mapping the project’s progress in detail, ensuring strategic direction with the participation of all those involved. The development phase, part of the information obtained from the previous phase, transforms them into technical details for production. And the post-development phase is monitoring the product on the market, identifying needs or opportunities for improvement^(13)^.

In the product pre-development phase, a work plan was carried out, where a detailed schedule of the steps was prepared, research was carried out in databases and a list of necessary requirements to construct the mask project for protection from surgical smoke, based on the following technical standards: nonwovens for articles for dental, medical and hospital use; nonwoven for articles for dental-medical-hospital use - determination of bacteriological filtration efficiency^(14)^; non-woven articles for dental-medical-hospital use - surgical masks – requirements^(15)^; respiratory protective equipment – particulate filtering half facepiece^(16)^.

To develop the prototype, multidisciplinary work was necessary, as it requires a complex process, demanding the vision of different areas of knowledge, such as nurses, chemists, specialists in toxicology and specialists in product design.

Therefore, to elaborate the prototype design of a mask to protect against surgical smoke, an industrial design office was hired.

All the information for developing this technological innovation, being a mask prototype, was obtained through research in the literature, and the results of research carried out previously by the Management, Scientific Publishing and Occupational Health Study Group (GeeST) were taken into account.

## RESULTS

This research aimed to develop a technological innovation for greater protection and safety for workers to develop their work activities as well as to present the characteristic of an individual, ergonomic, sustainable and effective product.

Thus, activities were initiated in the first step, Sense Intent, for compiling information and opening the project. With the information collected, the necessary requirements for developing the prototype were listed, described in Chart 1.

**Chart 1:** Necessary requirements for developing a technological innovation of a mask prototype for protection against surgical smoke

Product requirements	Attribute	Specification	Necessary or desirable
Material must allow cleaning by autoclaving	Safety	Suggestion: silicone	Necessary
Being adaptable to different face sizes	Comfort and safety	Being of malleable/adaptable material	Necessary
		Having size options (S, M and L)	Desirable
		It must have adjustment in the fastening strap	Desirable
Allow use for long periods without causing harm to the user’s face	Comfort	Being of malleable/adaptable material	Necessary
		Fixation must be on the head and not on the ears	Necessary
		Higher pressure points on the face should be comfortable	Necessary
Allow communication between professionals	Usability	Material must not insulate sound	Desirable
It must be single use and reusable	Usability	Must have disposable filter	Necessary
Product should not scare patients	Aesthetic	Must not have exhalation valves	Necessary
About detachable parts (ABNT NBR 13698:2011 standard)	Safety	All detachable parts (if any) must be easily connected and held securely in the part without the use of tools.	Necessary
About finishing (ABNT NBR 13698:2011 standard)	Comfort	The finish of any part of the mask that comes into contact with wearers must be free of burrs or sharp edges.	Necessary
Mask must seal completely on the face	Safety	No openings between the mask and the user’s face	Necessary
It should be under the chin	Safety	Keeping the seal.	Necessary

After describing the necessary requirements for the development of the mask, the Know Context and Know People phases began, where market and people research was carried out to understand the context and users. Knowing people is to acquire understanding of thoughts, needs and feelings, through observation, interaction and analysis of their daily life and routine, revealing perceptions, valuable insights^(12)^. In this regard, it was possible to identify potential insights to be used in the ideation and solution phases.

**Figure 1:**
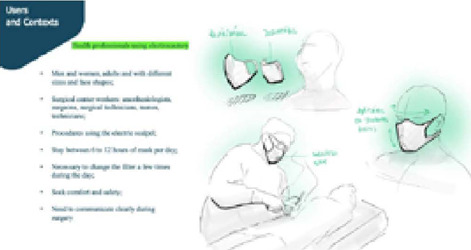
People, Objects, Environment, Message and Services (POEMS) tool for use in the development of surgical smoke protection mask

Market research was carried out in order to seek information for product planning. Several masks available on the market were also purchased, both for use to protect health personnel and industrial workers. In the literature, the pressure points of faces and types of faces were verified. For this purpose, the tool People, Objects, Environment, Message and Services (POEMS) was used, shown in Figure 1.

**Figure 2:**
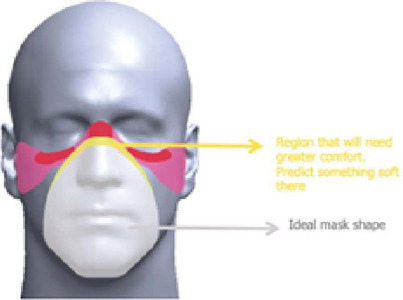
Study of pressure points on users’ face for the development of a surgical smoke protection mask prototype

Next, in Figure 2, the study of pressure points on the face of users is presented.

After studying the faces, a simulation was performed of use of masks purchased on the market in general, by the researchers, to verify each mask’s strengths and weaknesses, and thus provide subsidies for developing the prototype.

In the previous steps, the context and users’ understanding were discussed, obtaining knowledge and information about the object of study, after which the Frame Insights phase began, where the collected information was understood, transforming it into useful and concrete interpretations for prototype development. Based on this information and analysis of the requirements listed for developing the prototype, the Explore Concepts phase was started, where the project solutions were defined, and then proceed to product creation. It is the phase known as ideation, which is the moment when the team creates ideas centered on users and on the context studied in the previous steps^(12)^.

This step started with Principles to Opportunities, in which the observed insights provide a good structure to move from understanding to definition and exploration to generate concepts. It is a method in which the transition from the Frame Insights phase to the Explore Concepts phase is done in a disciplined manner so that concepts are grounded in objective research data rather than biased by subjective assumptions^(12)^. Thus, the design of the material to be developed began, going through all the necessary phases for its elaboration.

**Figure 3:**
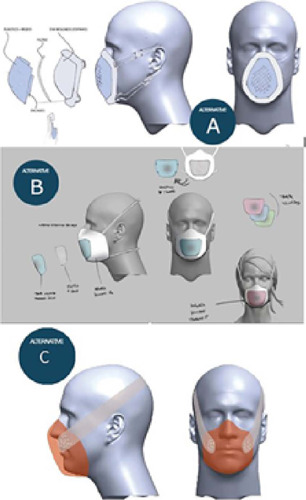
Design suggestions for mask prototype for protection against surgical smoke

Initially, a pattern was created and some suggestions were made based on the information collected, as shown in Figure 3.

Suggestions were in line with the insights generated and the requirements raised in the information gathering phase. Thus, alternative B was chosen as a viable proposal to achieve the objective proposed in this study. From this choice, a prototype model was created, with alternative materials, to encourage intended functions, through observation and user experience, to validate or invalidate the proposed solutions. Information was collected through observation and user interaction with the prototype through use simulation, and recorded by photos and notes.

From the prototype model, where it became possible to represent the abstract to the physical, it was possible to implement the concepts created in the previous steps and make the necessary adjustments for creating a prototype model, which will present the concept for product commercialization.

The mask is presented in sizes S, M and L, according to face study and dimensions published in the literature^(17)^; however, for constructing the prototype, presentation is in size M, because it is the best suited to the analyzed faces. The prototype presented all the requirements selected and assessed throughout the development process.

From the prototyping mold, where it became possible to represent the abstract to the physical, the concepts created in the previous steps can be implemented and make the necessary adjustments for creating a prototype model in which there will be the concept for product commercialization with its sizing, shape and components.

Firstly, the model had a dimension that, after making the first impression of the prototype, it was verified that it needed adjustments in the field where the filter is placed, increasing the dimensions.

**Figure 4:**
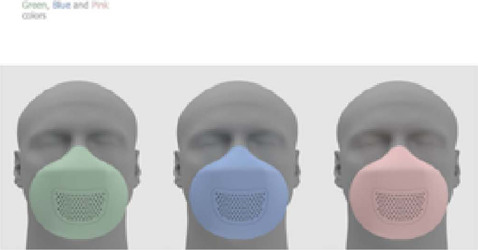
HeLP, a mask for occupational respiratory protection for workers exposed to surgical smoke

After all the steps for developing a technological innovation product, HeLP was created, a mask for occupational respiratory protection for workers exposed to surgical smoke, as shown in Figure 4.

## DISCUSSION

With this study, a mask was developed for respiratory protection of workers exposed to surgical smoke, thus avoiding the possible development of diseases linked to this chemical risk.

The development of new products that will serve the market or that will generate value for society is called innovation, understood as the search for novelty through new products, services or production methods^(18)^.

In this context, the product is understood as a set of palpable characteristics, which can be consumed by the market, meeting its needs^(18)^. HeLP protects workers who are exposed to the hazards that surgical smoke poses, such as inhaling toxic chemicals, in addition to being a product that could meet the market’s needs, since the masks available on the market are not manufactured for this type of protection.

The process of developing a product is characterized by a systematic process that goes through some steps to reach the objective^(18)^. Among these steps we have the prototyping of the product, which is the step in which the idea passes from the abstract to the physical. Through the prototype, project uncertainties are eliminated so that the final idea solution is assertive^(12)^.

As for the material used for mask prototype development, it was decided to use thermoplastic polyurethane (TPU), which is a more malleable filament, being the one that is closest to the silicone material, material to be used to make the mask. With TPU, it can be verified that the necessary requirements listed by the researchers have all been covered.

Once ready, HeLP fits the researchers’ face well, covering the entire chin and sealing the face. The filter cover is sized for ease of breathing, and this cover fits easily onto the mask.

For the tests suggested for validating the mask, the next step of the study, It will be made of silicone or resin material, and will have an activated carbon filter, as it is capable of adsorbing the aromatic polycyclic hydrocarbons present in surgical smoke^(7)^, in addition to being able to filter up to 95% of particulate matter.

An important point in the development of the protective mask is that the material chosen for finalization was silicone or resin that will allow disinfection and/or sterilization, therefore it will be reused, contributing to sustainability and reduction of waste generated in health institutions.

The development of a new technology and innovation product is a complex process, therefore, as expected, the last step will be carried out directly on the operating room workers.

### Study limitations

It is known that, for the development of a product, a complex process is necessary that demands study, time, effort, dedication and financial resources. To develop and manufacture a mask prototype, financial resources from the researcher herself were used. Therefore, it was not possible to make the product in appropriate material and validate it, such as breathability, sealing and comfort tests.

### Contributions to nursing, health, or public policies

It is expected that the product generated in this research will protect workers who are in contact with surgical smoke, in order to prevent the development of diseases linked to this risk. Furthermore, a more comfortable protective mask can be more easily adhered to by exposed workers, in addition to being sustainable and allowing for more time of use by the same worker.

This advance in science is necessary so that managers and workers can act together to protect the health of those exposed, in the sense of making choices regarding PPE, making its implementation reliable and adequate for those involved.

## CONCLUSIONS

This study describes the development process of HeLP, an occupational protection mask against surgical smoke, from the design step to the prototype development for subsequent testing and manufacture of the definitive mask to protect workers exposed to surgical smoke.

It is stated that it is possible, with the involvement of science, to develop a mask for the occupational protection of workers exposed to surgical smoke for individual use, subject to disinfection, ergonomic and sustainable, thus being able to increase compliance with its use, protecting users from the development of diseases linked to this exposure.
